# Supplementary material for: Characterization of Novel Human β-glucocerebrosidase Antibodies for Parkinson’s Disease Research
Source: J Parkinsons Dis. 2024 Jan 23;14(1):65–78. doi: 10.3233/JPD-230295 (PMC10836542; doi:10.3233/JPD-230295)

Supplementary Material

Characterization of Novel Human β-glucocerebrosidase Antibodies for Parkinson's Disease Research

Supplementary Figure 1. Characterization of hGCase-1/17 and hGCase-1/23 hybridoma clones. Hybridoma clones hGCase-1/17 and hGCase-1/23 were derived from mice immunized with imiglucerase. HGCase-1/23 demonstrated stronger potency than hGCase-1/17 towards hGCase overexpressed in HEK293-F cells in western blotting and in immunofluorescence assays. Neither of the two antibodies cross-reacted with mouse β-glucocerebrosidase (mGCase) overexpressed in HEK293-F cells, demonstrating their species specificity toward hGCase.

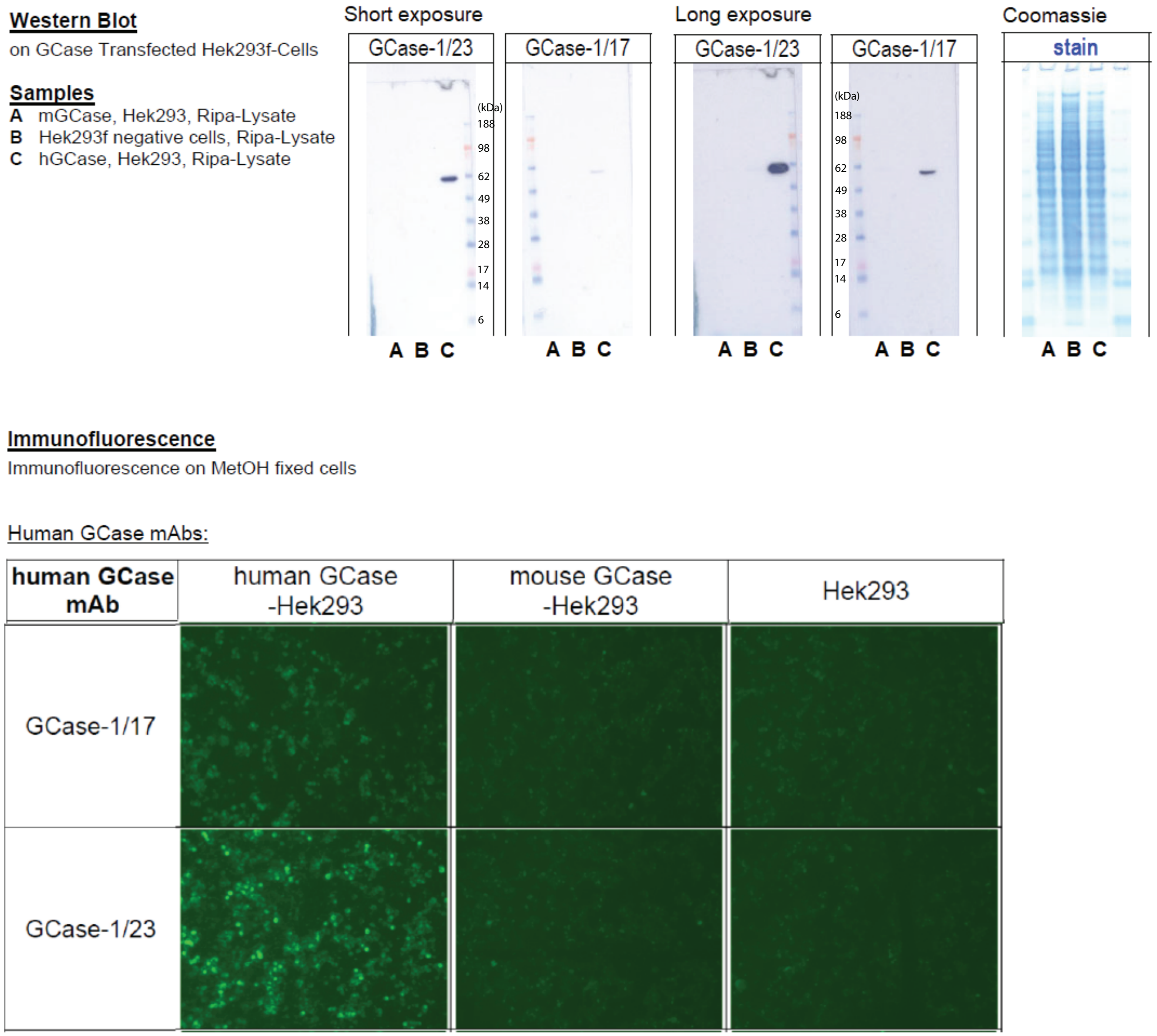

Supplementary Figure 2. Frameshift indels in the two *GBA1* alleles targeted by ZFN but lacking resistance cassette integration. Sanger sequencing *GBA1* alleles without resistance cassette integration showed double peaks in the chromatogram indicating the two *GBA1* alleles were targeted by ZFN as well. Parsing the double peak chromatogram using Poly Peak Parser revealed frameshift indels in both alleles.

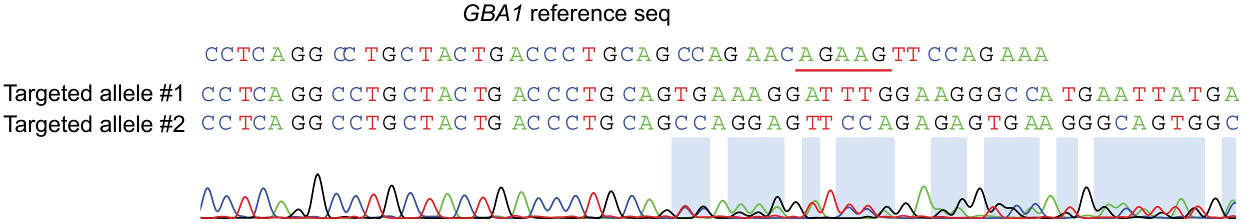

Supplementary Figure 3. No specific immunostaining of hGCase in H4 cells with hGCase antibody 812201. *GBA1* +/+ H4 cells were fixed with 4% PFA, permeabilized with 0.05% Saponin, and stained with hGCase antibody 812201, together with LAMP1 antibody. The staining pattern was diffusive showing no localization in lysosomes marked with LAMP1. Scale bar: 10 μm.

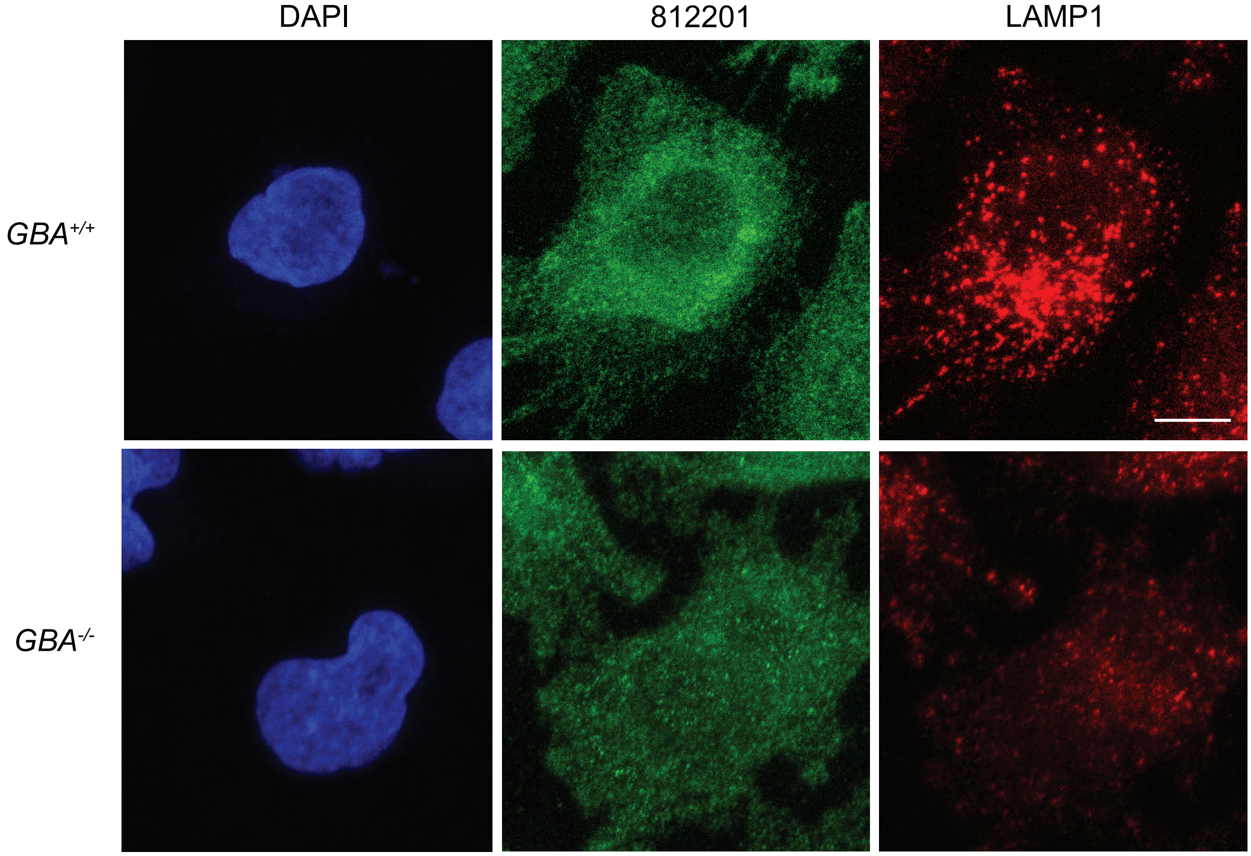

Supplement: Supplementary Material [file jpd-14-jpd230295-s001.pdf]
